# Abamectin Efficacy on the Potato Cyst Nematode *Globodera pallida*

**DOI:** 10.3390/plants9010012

**Published:** 2019-12-19

**Authors:** Nicola Sasanelli, Ion Toderas, Pasqua Veronico, Elena Iurcu-Straistaru, Stefan Rusu, Maria Teresa Melillo, Pierluigi Caboni

**Affiliations:** 1 Institute for Sustainable Plant Protection, CNR, Via G. Amendola 122/D, 70126 Bari, Italy; pasqua.veronico@ipsp.cnr.it (P.V.); mariateresa.melillo@ipsp.cnr.it (M.T.M.); 2 Institute of Zoology, MECC, St. Academiei 1, 2028 Chisinau, Moldova; iontoderas@yahoo.com (I.T.); iurcuelena@mail.ru (E.I.-S.); rusus1974@yahoo.com (S.R.); 3 Department of Life and Environmental Sciences, University of Cagliari, Via Ospedale 72, 09124 Cagliari, Italy; caboni@unica.it

**Keywords:** bio-pesticides, *streptomycetes*, nematode control, plant parasitic nematodes, nematicide

## Abstract

The potato cyst nematode *Globodera pallida* is a major pest of the potato crop. Abamectin is a biological pesticide showing high nematicide activity, but its efficacy to control *G. pallida* has not been investigated to date. In this study, combination of different abamectin concentrations ranging from 1.125 to 36 µg/mL x exposure times from 24 to 384 h were tested on the nematode in a hatching test. Abamectin induced mortality with LD_50_ value in the range of 13.23 (after 24 h) to 2.90 µg/mL (after 384 h). A glasshouse experiment was also performed in pots filled with soil infected with *G. pallida* in the presence of sprouted potato tubers cultivar “Spunta”. Abamectin at 4.5, 9.0, 18.0 and 36.0 µg/mL was used in comparison with nematicide fosthiazate. The doses of 18 and 36 µg/mL significantly reduced number of eggs, juveniles, cyst/g soil and reproduction rate in comparison to both untreated control and fosthiazate treatment. Soil applications of abamectin provided significant *G. pallida* control with LD_50_ and LD_99.9_ of 14.4 and 131.3 µg/mL, respectively. These results indicate the efficacy of abamectin to control *G. pallida* on potato crops and its potential use in organic agriculture or in an integrated pest management program.

## 1. Introduction

Potato (*Solanum tuberosum* L.) is one of the most important food crop in the world and it is grown in more than 100 countries under temperate, subtropical and tropical conditions. Many factors can affect potato growth including plant parasitic nematodes. The potato cyst nematode (PCN) *Globodera pallida* (Stone) Behrens is among the most devastating plant parasitic nematodes causing severe yield losses in many potato growing areas of the world (CABI datasheet) [1]. The extent of yield loss is related to soil nematode population density at sowing which should be always maintained below the estimate damage threshold (1.7 eggs and juveniles/g soil) [2]. *Globodera pallida* is difficult to manage because there are currently few genetic resistance sources available in commercially-grown potato cultivars [3]. Moreover, *G. pallida* populations are able to completely overcome the resistance of cultivars carrying the GpaV_vm_ in only few generations [4]. Potato cyst nematodes are routinely controlled using nematicides in many temperate countries [5,6]. However, the use of pesticides on agricultural crops has been deeply revised and restricted by the European Legislation (Reg. EC 1107/2009; 459/2010 and 293/2013). Therefore, attention has been focused on alternatives to chemicals at low environmental impact [7,8,9,10,11,12]. An interesting alternative is the use of bio-pesticides which are generally based on the direct effect of fungi and bacteria or their exametabolites [13,14]. *Streptomyces* spp. are Gram-positive filamentous actinobacteria that are well adapted to the soil environment. They produce a wide range of biologically active substances especially antibiotics and hydrolytic enzymes that protect plant growth from pathogenic fungi and bacteria [15,16,17]. Fermentation of products derived from *Streptomyces avermitilis* produces macrocyclic lactones, the avermectins (AVM) with insecticidal, acaricidal and nematicidal properties [18]. Compounds derived from avermectin that show great biological activity as anthelmintic agents include abamectin, doramectin, ivermectin and selamectin. In particular, abamectin is a mixture constituted roughly of 80% of avermectin B1a and 20% of avermectin B1b [19]. Avermectins block the γ-amino butyric acid stimulated chloride channels and open non-neurotransmitter-gated chloride channels [20], causing an ion imbalance in the nervous system, resulting in paralysis. Abamectin is registered as an acaricide/insecticide in more than 50 countries. This compound is important in animal health and crop protection [21,22,23]. It can be stored for many months maintaining its biological properties and it can be also used as seed treatment to protect plants, in the initial growing phase, from plant parasitic nematodes and insects which attack root systems [24]. Abamectin has a low water solubility and is bound tightly to soil particles, resulting in poor movement of the product through the soil profile and its leaching potential through different types of soil is extremely low depending from soil characteristics, oxygen content and microbial activity [25,26]. Abamectin is known to have nematicidal activity against some plant parasitic nematodes as the root lesion nematode *Pratylenchus penetrans* [27], the reniform nematode *Rotylenchulus reniformis*, the root-knot nematode *Meloidogyne incognita* [28,29,30] and the cyst nematodes *Heterodera schachtii* [31], *H. avenae* [32] and *H. carotae* [33]. In 2015 it was registered in the EU as a nematicide against root-knot nematodes with the commercial name Tervigo SC on tomato, eggplant, pepper and string-beans. Although the nematicidal effect of abamectin is well known, no information is available on its effect on the PCN *G. pallida.*

The aim of this work was to test the nematicidal efficacy of an abamectin formulation (Vertimec^®^ EC) on the PCN *G. pallida* in vitro and to determine the effective dose for its control in a glasshouse experiment as a preliminary approach to field application. 

## 2. Results and Discussion

### 2.1. In Vitro Test on Cysts

The nematicidal effect of abamectin treatments on *G. pallida* cysts is reported in Table 1. 

Hatching of second-stage juveniles (J2) was significantly lower than that observed in the untreated control already after 24 and 48 h exposure at all the tested rates, with the exception of 1.125 μg/mL of abamectin aqueous solutions (*P* < 0.01). Hatching percentage at the highest abamectin concentration (36 μg/mL) was the lowest recorded. At 96 h the percentages of hatching at the two highest concentrations (18 and 36 μg/mL) were significantly lower (10.0% and 6.4%, respectively) compared to the control and they were not significantly different from each other (*P* < 0.05). At longer exposure time (192 h) hatching from cysts treated with the three highest abamectin concentrations (9, 18 and 36 μg/mL) was significantly lower than those in the control and in the two lowest abamectin concentrations (*P* < 0.01). At 384 h exposure, hatching from cysts treated with 4.5 μg/mL abamectin solution was also significantly reduced (*P* < 0.01). At each concentration value data analysis clearly indicated a progressive significant reduction of the hatching percentages over time starting from 96 h exposure (*P* < 0.05). The fall in hatching numbers in the control as exposure time increased (384 h) could be attributed to the permanence of cysts in water which may have affected the eggshell permeability and the eclosion [34].

The factorial analysis showed significant (*P* < 0.01) effect of both factors abamectin concentration (Factor A) and exposure time (Factor B) and of their interaction A×B on hatching (Table 1). Overall, these data indicated that increasing abamectin concentration and exposure time significantly decreased hatching percentages. Hatching was reduced of about 50% when using at least 9 μg/mL of abamectin for 24 h. At the longest exposure times, lower abamectin doses were required to obtain a similar percentage of hatching reduction. The in vitro experiment indicated the strong nematicidal effect of the abamectin aqueous solution on *G. pallida*.

Table 2. shows the mortality percentages calculated on the basis of the unhatched eggs which are not viable [35]. The mortality of *G. pallida* increased at each exposure time increasing the abamectin concentration. On the other hand, at the same concentration an increase of mortality was observed by the increase of exposure time, except for the lowest concentration (Table 2). A mortality higher than 50% was observed only when cysts were exposed for 24 and 48 h at the highest concentration. 

By increasing the exposure time, a lower abamectin concentration was required to obtain 50% mortality (Table 2). This result is important for the reduction cost of nematode management. According to probit analysis the LD_50_, although with some variations, was in the range from 13.2 (after 24 h exposure) to 2.9 μg/mL (after 384 h exposure) (Table 3). Moreover, doses in μg/mL to achieve greatest mortalities (60, 70, 80, 90 and 99.9%) were in the range of 23.1–4.0, 42.3–5.7, 86.4–8.7, 230.8–15.5 and 2408.2–61.3, at the least (24 h) and longest (384 h) exposure times, respectively (Table 3). The values of LD_50_ obtained were higher than those reported for *H. avenae* where J2 were directly exposed to abamectin solution [32]. This result suggests that cyst cuticle works in some way as a barrier to the bio-pesticide. Moreover, in our study, LD_50_ values were also higher than those found for the carrot cyst nematode *H. carotae* in the same experimental conditions (concentrations x exposure times) [33] demonstrating a lower sensitivity of *G. pallida* to abamectin exposition than *H. carotae*. 

### 2.2. Abamectin Effect on Viability and Infectivity of G. pallida J2

**Figure 1:**
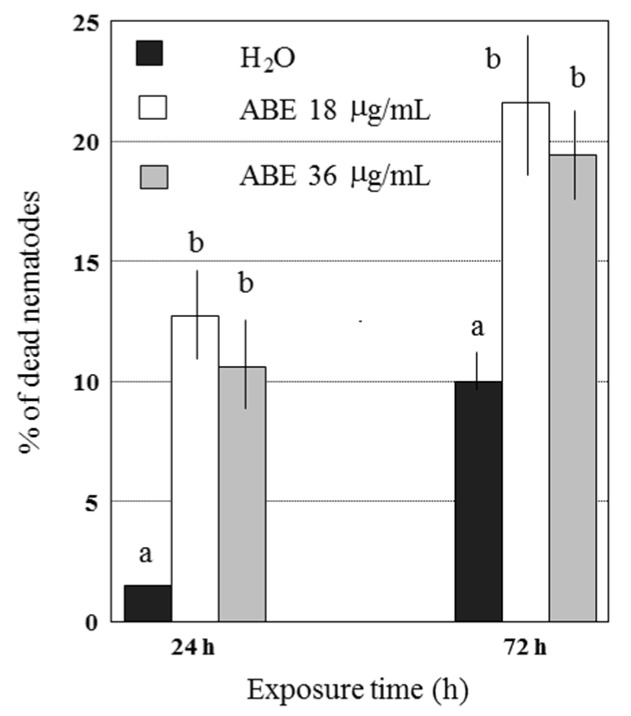
Effect of two abamectin concentrations, 18 and 36 μg/mL, on the per cent mortality of second stage juveniles of *Globodera pallida*. Data are means ± SE from two experiments in which 6 batches of *G. pallida* J2 per each treatment were used. Above each column and for each exposure time different letters indicate significant differences according to Least Significant Difference test (*P* ≤ 0.01).

In addition to the lethal effect of abamectin on *G. pallida* eggs, we evaluated its direct effect on J2, the infective stage of the nematode. The concentrations more effective (18 and 36 μg/mL) in killing more than 50% of eggs were tested. After 24 h incubation, nematodes exposed to both abamectin solutions showed significant differences (*P* < 0.05) in mortality (about 12%) compared to the untreated control in which 1% natural mortality was observed (Figure 1). No difference was evident between the two abamectin concentrations. After a 72 h incubation time, mortality of 21% and 19% were recorded at 18 and 36 μg/mL concentrations, respectively, compared to the control (9%) suggesting a nematicidal effect on *G. pallida* J2 (Figure 1). 

**Figure 2:**
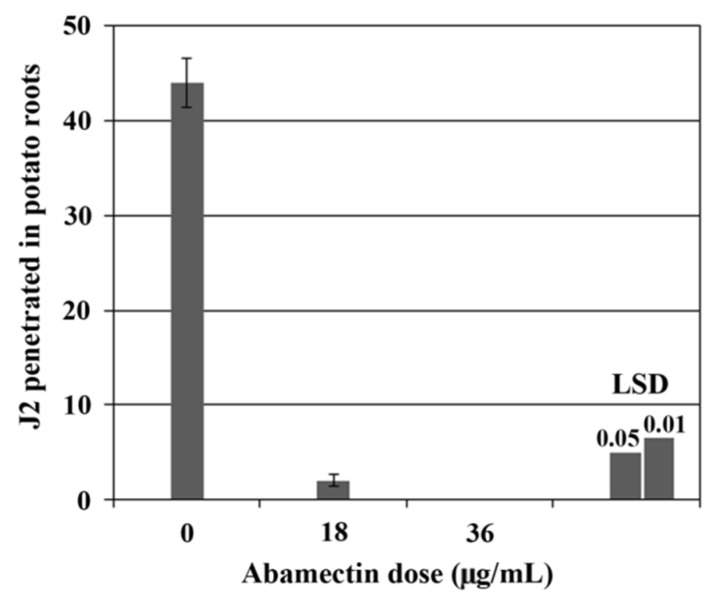
Root invasion assays. Number of *Globodera pallida* juveniles detected in potato roots (cv. Spunta) at 14 days after inoculation (dai). J2 were treated with different abamectin concentrations for 24 h before the inoculation. Data are means ± SE from two experiments each containing 5 plants per each treatment.

To verify whether abamectin can hamper the infectivity of nematodes, J2 previously incubated for 24 h in 18 and 36 μg/mL of abamectin solutions were inoculated on potato roots. At 14 days after inoculation (dai) the observation of potato root systems revealed that 24 h-treated juveniles were not able to penetrate the roots (Figure 2). Same results were obtained with 72 h-treated nematodes (data not shown). 

In vitro and *in planta* tests showed that abamectin negatively influenced viability and infectivity of *G. pallida* J2 when applied for 24 and 72 h at 18 and 36 μg/mL rates. The exposure time of 24 h induced a reduction in nematode viability (−12%) as previously reported for *M. incognita* and *R. reniformis* [28] thus confirming abamectin nematotoxic effect. Nevertheless, juveniles showed a recovery from paralysis when removed and rinsed from abamectin, but they were too weak to infect potato roots. It has been reported that lipid stores are implicated in the infective potential of plant parasitic nematodes [36]. We can hypothesize that the observed loss of infectivity could be the effect of abamectin on depletion of endogenous lipid reserves used for motility and root invasion as previously observed for *G. pallida* J2 treated with ivermectin [37]. However, these findings could be consistent with a multi-level effect of macrocyclic lactones which led also to an inhibitory action on nematode neuromuscular function [37].

### 2.3. Glasshouse Experiment

Based on the results obtained in in vitro test, experiments in controlled conditions were carried out to confirm the efficacy of the different doses when applied in the presence of the host plant and soil. A glasshouse experiment was set up to obtain preliminary information about doses of abamectin to apply in field condition. Pot experiment and a hatching test on newly formed cysts, were undertaken at temperature of 20 °C suitable for *G. pallida* reproduction. The abamectin was applied two weeks after sowing when the emission of young roots from sprouted potatoes produced radical exudates which allowed emergence of J2, so protecting immediately the crop from the nematode attack. In Table 4 significant reductions of *G. pallida* cysts per 100 g dried soil in the two highest abamectin concentrations (18 and 36 μg/mL) and in the fosthiazate treated pots were observed in comparison to the untreated control and the two lowest abamectin doses. A similar trend was observed for the number of eggs and J2 per g dried soil. The lowest numbers of eggs and J2 were observed in soil treated with 18 and 36 μg/mL abamectin aqueous solution (*P* < 0.01) and they were about ten times lower compared to the control (Table 4). These treatments were more effective than fosthiazate. Likewise, the number of eggs and J2 per cyst was also significantly affected by the different treatments. The soil application of abamectin aqueous solutions at 18 and 36 μg/mL significantly reduced number of eggs and J2 per cyst (−48% and −37%, respectively) compared to the control. No statistical difference was observed between abamectin at 36 μg/mL and fosthiazate treatments (*P* < 0.01) (Table 4).

**Figure 3:**
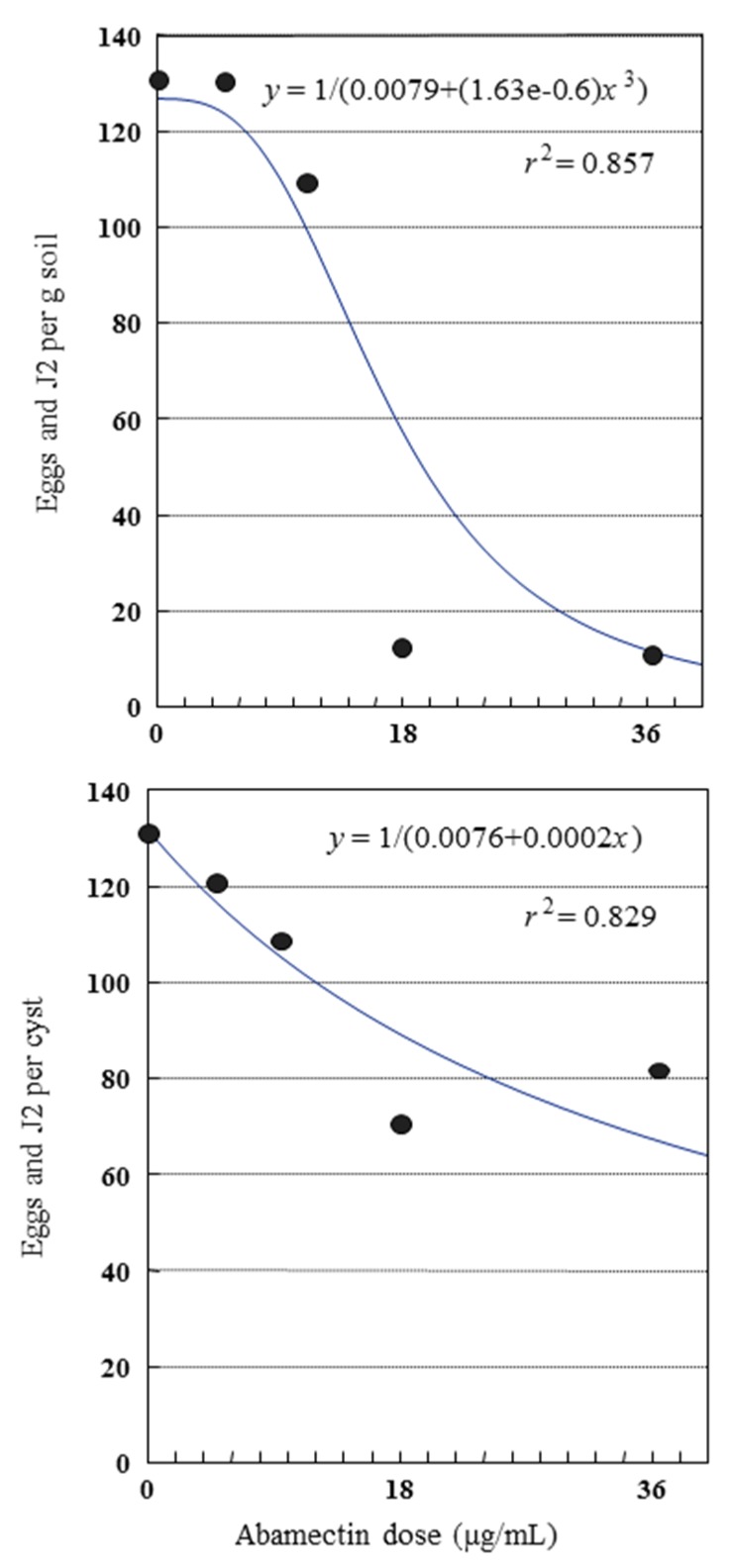
Relationship between *Globodera pallida* eggs and juveniles per g soil and/or cyst and applied abamectin dose. Each data point represents eggs and J2 at 0, 4.5, 9, 18 and 36 µg/mL abamectin, calculated from the mean of 16 replications from two independent experiments. Equations for regression lines were calculated from the experimental points.

The highest *G. pallida* reproduction rate (4.6) was recorded in the control and in the soil treated with 4.5 μg/mL abamectin aqueous solution. A significant lower reproduction rate (0.5) was observed in both highest abamectin doses (*P* < 0.01) (Table 4). Reproduction rate at these doses was also significantly lower than that in fosthiazate (1.5) (Table 4). These results showed that the two highest abamectin doses were more effective in the control of nematode infection than the chemical nematicide. A significant negative correlation index was found for both relationships between eggs and J2 per g dried soil and/or per cyst and the abamectin applied doses (Figure 3).

In the soil the dose to kill 50% (LD_50_) of eggs and J2 per g dried soil was 13.8 μg/mL according to the equation *y* = −0.078 + 4.457*x* in which *y* is the % mortality in probit values and *x* the dose, expressed in μg/mL. Therefore, the need to use a higher dose in pot experiment (18 μg/mL) *versus* in vitro hatching test (9 μg/mL) could be due to abamectin dispersion through the soil which limited the direct contact of the bio-pesticide with the cysts [26,38]. At the abamectin rate of 18 μg/mL, number of cysts, eggs and J2 per g dried soil and per cyst and reproduction rate were strongly reduced of −79.5, −89.7, −47.7 and −89.1%, respectively, compared to the control. Interestingly, abamectin was more effective in controlling *G. pallida* infection on potato than fosthiazate which reduced the above mentioned parameters of −59.2, −68.2, −23 and −67.4%, respectively.

Since the degree of efficacy of potato management varies, especially in terms of per cent yield increase under different nematode pressure levels, the effect of abamectin on fecundity of newly formed cysts and vitality of eggs within them must be investigated. Indeed, *G. pallida* cysts with viable eggs can persist for several years in the absence of a suitable host. The encysted eggs remain dormant until infective juveniles hatch from them in response to root diffusate from potato plants [34]. Therefore, a hatching test was carried out to analyze the vitality of eggs inside the new formed cysts derived from the pot experiment. Data showed a significant reduction for almost all the concentrations used (Table 5). The highest significant reduction was recorded in cysts from soil treated at the 36 μg/mL abamectin dose. Moreover, percentage of hatching observed in these cysts was significantly lower (4.6%) than that from soil treated with fosthiazate (28.0%). The highest abamectin dose allowed a significant reduction in J2 hatching compared to the untreated and fosthiazate-treated cysts (−91 and −84%, respectively), thus resulting the most effective treatment to prevent damages to the following potato crop. On the bases of tolerance limit (*T* = 1.7 eggs and J2/g soil) and the minimum relative yield (*m* = 0.03) for *G. pallida* and potato [2] it was possible to estimate the yield loss for the following potato crop according to the modified Seinhort’s equation [39] *y* = *m* + (1 − *m*) 1.05^[(*P*/−*T*)+1]^, describing the relationship between relative yield (*y*) and soil nematode population density (*P*). Without any soil treatment the expected yield loss based on number of eggs and J2/g soil (Table 4) and hatching percentage (Table 5) was 80%. Conversely, treatment with the highest abamectin dose determined an infecting rate of 0.64 eggs and J2/g soil (14 × 4.6/100) lower than the tolerance threshold with no impact on yield. Abamectin resulted more effective in *G. pallida* management than fosthiazate for which a yield loss of 23% was calculated for the following potato crop.

Probit analysis applied to the hatching test on cysts collected from soil treated with different abamectin doses allowed to calculate the doses required to kill 50, 60, 70, 80, 90 and 99.9% egg vitality. The doses were 14.4, 17.2, 20.9, 26.3, 36 and 131.3 μg/mL, respectively (Table 6). Glasshouse pot experiments are predictors of potential abamectin efficacy in field conditions. 

## 3. Materials and Methods

### 3.1. Globodera Pallida Population 

A population of *G. pallida* was extracted from soil samples collected in a heavily infested field at Conversano (40°57’12” N, 17°09’22” E; Southern Italy). Cysts were extracted from each sample by the Fenwick can [40] for nematode identification. Potato cyst nematodes were identified as *G. pallida* on the basis of morphometric measurements such as shapes of cuticular ridges and juvenile’s stylet length [(EPPO PM 7/40 (4)] [41]. Subsequently, *G. pallida* was classified as pathotype Pa3 on the basis of multiplication on different *Solanum* clones [42].

*Globodera pallida* was reared on potato (*Solanum tuberosum* L.) cv. Spunta for 90 days in a glasshouse at 20 ± 2 °C to obtain cysts to be used in all the experiments. 

### 3.2. In Vitro Tests on Cysts

A commercial formulation, Vertimec® EC (Syngenta Italia S.p.a., Milan, Italy), based on abamectin (18 g/L) solubilized in N-Methyl-2-Pyrrolidone, at different concentrations [(0, 0.0625, 0.125, 0.25, 0.5, 1 and 2 mL/L of commercial product (c.p.) corresponding to 0, 1.125, 2.25, 4.5, 9, 18, and 36 μg/mL of active ingredient (a.i.)] was tested against *G. pallida* at different exposure times (24, 48, 96, 192 and 384 h). The different concentrations in a geometric series, were obtained diluting a stock solution (2 mL/L) of the Vertimec® EC in distilled water.

Batches of 50 brown cysts of similar size (averaging 7530 eggs per batch) were placed on 2 cm diam sieves (215 μm aperture). Each sieve was put in a 3.5 cm diam Petri dish, and all dishes were arranged in a completely randomized experimental design. Three mL of test solutions containing abamectin at the established concentrations were added to *G. pallida* cysts. The treatments (concentration × exposure time combination) were replicated three times. The experiment was performed twice. After treatments, cysts were removed from abamectin solutions, rinsed in distilled water and incubated in the artificial hatching agent 0.6 mM sodium metavanadate aqueous solution [43] in a growth cabinet at 20 °C for hatching. Batches with untreated cysts, exposed only to distilled water, were used as control. Emerged juveniles were removed and counted every week, renewing the hatching agent at the same time, over an eight weeks period. At the end of the hatching test, cysts were crushed with the Bijloo’s modified method [44] and unhatched eggs and juveniles were counted. Emerged juveniles and unhatched eggs (hatched + unhatched eggs) were considered as total numbers of eggs per replicate at the beginning of the experiment. Hatching rate was calculated as the per cent ratio between number of emerged juveniles and total number of eggs and difference to 100 was assumed as mortality rate [35]. The percentages of mortality were corrected by eliminating the natural death in the distilled water control according to the Schneider-Orelli’s formula [45]:

Corrected mortality % = [(Mortality % in treatment − Mortality % in the control) / (100 − Mortality % control)] × 100.

Data on mortality percentage were subjected to probit analysis [46] to estimate the values of lethal doses (LD), e.g., the abamectin dose required for 50, 60, 70, 80, 90 and 99.9% egg mortality at each exposure time.

### 3.3. Abamectin Effect on Viability and Infectivity of G. pallida J2

One hundred J2 of *G. pallida* were placed into a 3.5 cm diam well in a 6-well culture plate containing 1 mL of 18 and 36 μg/mL abamectin aqueous solution or 1 mL of distilled water as control treatment. The lowest abamectin concentrations (4.5 and 9 µg/mL) were not considered since they were not able to kill more than 50% of eggs at the exposure times considered (24 and 72 h) in the in vitro test on cysts. All samples were incubated in the dark at 20 °C for 24 h and 72 h. At the end of incubation, 3 drops of 1N NaOH were added to each well. Non-motile J2 that reacted to NaOH by changing their body shape within 3 min were considered alive, whereas straight nematodes that failed to respond were classified as dead [47]. The alive and dead J2 were counted under a stereo microscope and percentages of mortality calculated. The experiment was performed two times with six replicates for each treatment in a completely randomized experimental design. To verify the effect of abamectin on juvenile’s infectivity, freshly hatched *G. pallida* J2 were incubated, for 24 h and 72 h at 20 °C in the dark, in abamectin aqueous solutions at 18 and 36 μg/mL and in distilled water for the control treatment. After incubation J2 were rinsed with water to remove abamectin. Then, 300 J2 were inoculated on one-month old potato seedlings (cv. Spunta) grown in pots containing 100 mL of sterilized sand in a growth chamber (12 h of light and 12 h of darkness, 20 °C). At 14 dai, the root systems were collected and stained with acid fuchsin solution (0.013% acid fuchsin in 0.8% acetic acid) to detect nematodes inside the roots. Roots were boiled for 3 min in staining solution and after cooling at room temperature the excess liquid was drained. The roots were washed with running tap water and de-stained in acidified glycerol. Nematodes inside the roots were counted using a stereo microscope at 20x of magnification and their total number was recorded. The experiment was performed two times with five replicates for each treatment in a completely randomized experimental design.

### 3.4. Glasshouse Experiment

For the in vivo experiment *G. pallida* at initial population density (*Pi*) of 27 eggs and juveniles/g soil was used. Inoculum was prepared according to the procedure described by Renčo et al. [12]. Clay pots (12 cm diameter, 1 L) were filled with 1300 g dried infested soil. One potato tuber (cv. Spunta) was sown in each pot. All pots were arranged on benches in a glasshouse at 20 ± 2 °C in a randomized complete block design with eight replications for each treatment. The experiment was performed twice. 

Treatments were (a,b,c,d) abamectin aqueous solutions at concentrations of 4.5, 9.0, 18.0 and 36.0 μg a.i./mL, (e) fosthiazate (nematicide, chemical control) applied at sowing as recommended in the product label at the field rate of 3 g/m^2^. Untreated pots were used as control. The abamectin concentrations were selected in a geometric scale to apply probit analysis for lethal doses calculation. Abamectin solutions were applied 2 weeks after sowing distributing 250 mL solution per pot obtaining in each pot of each treatment concentrations of 0.865, 1.73, 3.46 and 6.92 μg/g dried soil, respectively.

During the experiments, potato plants were maintained in the glasshouse randomizing the position of the blocks and at the same time repositioning each plant within a block every week, to avoid a block position effect as well as the factor position of the potato plant within the block. Plants were watered using the same amount of water per pot and fertilized every week with 100 mL of a 0.1%, 20-5-32 (N-P-K) micronutrients hydro-sol fertilizer solution (Haifa Chemical Ltd., Haifa, Israel). 

At the end of the experiment, three months later, when potato plants were dried, a 500-g soil sample was collected from each pot and cysts were extracted from 200 g dried soil by the Fenwick can. Cysts were counted and crushed and the number of viable eggs and J2 counted. Number of cysts per 100 g dried soil, eggs and J2 per cyst, eggs and J2 per g soil and the reproduction rates (*r* = *Pf*/*Pi*) were also calculated. 

In addition, a hatching test was set up, as described in Section 3.2, to verify the effect of soil abamectin treatments on the viability of eggs inside the newly formed cysts. Batches of 50 newly formed cysts of similar size were incubated to hatch in a 0.6 mM sodium metavanadate aqueous solution at 20 °C. For each treatment four replications were considered and the hatching test was performed twice. Juveniles emerged from eggs were removed and counted weekly over an 8-weeks period until no further hatch was observed. The hatching agent was renewed weekly. At the end of the hatching test, cysts were crushed, and unhatched eggs and juveniles were counted. Numbers of J2 emerging weekly were expressed as cumulative percentages of the total egg content of the cysts.

For both experiments, in pot and the hatching test, the percentages of reduction of the nematode population and hatch were calculated, respectively. Moreover, the percentages of mortality were calculated and corrected by eliminating the natural death in the control as previously described in Section 3.2.

### 3.5. Statistical Analysis

Data from hatching test, pot experiment and J2 viability and infectivity tests were subjected to analysis of variance (ANOVA) and means compared by Least Significant Difference’s Test (*P* < 0.05 and *P* < 0.01). Data from hatching test were previously transformed by Bliss’ Tables in arcsine square root percent hatching. The effect of abamectin concentrations, exposure times and their interactions were statistical analyzed by a 7 × 5 factorial design. Data of percentage mortalities obtained from pot and hatching test were subjected to probit analysis [46] to estimate values of abamectin lethal doses (LD) required for 50, 60, 70, 80, 90 and 99.9% nematode mortality. All statistical analyses were performed using the Plot IT program Ver. 3.2 (Scientific Programming Enterprises, Haslett, MI, USA). The relationship between eggs and J2s per g dried soil and per cyst and the abamectin applied doses was calculated by the software Table Curve 2D (Jandel, San Rafael, CA, USA).

## 4. Conclusions

The lack of effective nematode management products following the EU agricultural pesticide revision has raised a large demand for new innovative nematicides that can combine the nematicidal efficacy with environmental and human health safety. In this study the commercial product based on abamectin (Vertimec® EC), tested for the first time on *G. pallida* Pa3 both in in vitro and in glasshouse experiments, showed its strong efficacy in the management of this cyst nematode. Considering its moderate persistence in the environment, low toxicity to non-target beneficial organisms [48], the degradability by soil microorganisms, its poor leaching potential not resulting in ground water contamination, abamectin could be a potential bio-nematicide to use in Integrated Pest Management programs and organic farming. The effectiveness of abamectin to reduce viability of eggs inside cysts could help growers to reduce soil nematode population density for the following potato crop. Thus, it is possible to conclude that this product can be suitable in potato plant protection especially considering that no cultivars with high level of resistance to *G. pallida* are currently available. However, further studies need to be conducted to evaluate the efficacy of this bio-pesticide in field conditions.

## Figures and Tables

**Table 1:** Factorial analysis of different abamectin concentrations and exposure times on hatching of *Globodera pallida* juveniles.

Treatment	Concentration (g/mL) a.i.	Hatching Percentage					LSD	
		Exposure Time (h)						
		24	48	96	192	384	0.05	0.01
Untreated Control	0	30.0 ± 1.4 ^a^	23.8 ± 0.5	20.8 ± 2.9	22.0 ± 0.6	16.4 ± 0.6	4.7	6.8
Abamectin	1.125	26.7 ± 1.3	21.5 + 0.6	18.0 ± 1.8	17.3 ± 1.6	14.8 ± 0.9	4.1	5.9
Abamectin	2.25	22.1 ± 1.7	18.0 ± 1.5	14.8 ± 2.5	15.4 ± 1.3	8.1 ± 0.8	5.2	7.4
Abamectin	4.5	21.8 ± 1.5	15.9 ± 0.8	13.5 ± 1.6	9.9 ± 0.4	4.1 ± 0.1	3.3	4.7
Abamectin	9.0	15.3 ± 0.4	14.4 ± 0.3	12.7 ± 0.4	5.4 ± 1.0	2.6 ± 0.8	2.1	3.0
Abamectin	18.0	15.1 ± 1.4	14.5 ± 1.1	10.0 ± 0.7	5.2 ± 0.9	1.8 ± 0.8	3.2	4.5
Abamectin	36.0	9.3 ± 0.9	8.0 ± 0.9	6.4 ± 0.1	5.0 ± 0.5	1.1 ± 0.8	2.3	3.3
**LSD**	0.05	3.9	2.7	5.2	3.1	2.2	---	---
	0.01	5.5	3.7	7.3	4.2	3.1	---	---
ANOVA F values Factor A: Abamectin concentrations 137.2 **								
Factor B: Exposure times 127.6 **								
A × B 2.9 **								

^a^ Each value is an average ± SE of six replications from two independent experiments; ** = F values significant at *P* < 0.01.

**Table 2:** Percentage mortality of *Globodera pallida* at different rates of abamectin after a range of exposure times.

Abamectin Doses (g/mL) a.i.		Exposure Time (h)					LSD	
		24	48	96	192	384	0.05	0.01
1.125		11.0 ± 4.2 ^a^	9.7 ± 2.4	13.5 ± 6.6	21.4 ± 7.4	9.8 ± 5.3	18.0	25.7
2.25		26.3 ± 5.6	24.4 ± 6.1	28.8 ± 12.1	30.0 ± 6.1	50.6 ± 4.7	23.4	33.3
4.5		27.3 ± 5.0	33.2 ± 3.4	35.1 ± 7.6	55.0 ± 1.9	75.0 ± 0.4	13.9	19.8
9.0		49.0 ± 1.4	39.5 ± 1.2	38.9 ± 1.9	75.5 ± 4.7	84.1 ± 5.0	10.3	14.7
18.0		49.7 ± 4.6	39.1 ± 4.5	51.9 ± 3.4	76.4 ± 4.1	89.0 ± 4.9	13.7	19.4
36.0		69.0 ± 3.0	66.4 ± 3.9	69.2 ± 0.4	77.3 ± 2.3	93.3 ± 5.2	10.6	15.1
**LSD**	0.05	13.0	12.1	21.0	14.8	14.1	---	---
	0.01	18.2	16.9	29.4	20.7	19.8	---	---
ANOVA F values Factor A: Abamectin concentrations 73.8 **								
Factor B: Exposure times 30.1 **								
A × B 2.1 **								

^a^ Each value is an average ± SE of six replications from two independent experiments; ** = F values significant at *P* < 0.01.

**Table 3:** Abamectin concentrations needed to obtain 50, 60, 70, 80, 90 and 99.9% *Globodera pallida* mortality at the different exposure times.

Exposure Time (h)	Abamectin Concentrations (µg a.i./mL) Needed for Different % Mortalities					
	50	60	70	80	90	99.9
24	13.2 (10.1–17.3) ^a^	23.1 (17.7–30.3)	42.3 (32.3–55.4)	86.4 (66.0–113.1)	230.8 (176.2–302.3)	2408.2 (1838–3154.2)
48	18.1 (12.9–25.4)	33.9 (24.1–47.6)	66.7 (47.5–93.8)	148.9 (105.9–209.3)	449.0 (319.5–630.9)	6250.0 (4447.3–8783.3)
96	13.2 (9.8–17.8)	24.6 (18.2–33.1)	48.0 (35.6–64.6)	106.1 (78.7–142.9)	316.0 (234.5–425.7)	4271.6 (3170.5–5755.1)
192	4.5 (3.6–5.6)	7.5 (6.0–9.3)	12.8 (10.3–15.9)	24.2 (19.5–30.2)	58.3 (46.8–72.6)	474.6 (381.1–591.1)
384	2.9 (2.4–3.4)	4.0 (3.4–4.7)	5.7 (4.8–6.8)	8.7 (7.4–10.3)	15.5 (13.1–18.3)	61.3 (51.8–72.6)

^a^ Fiducial limits in brakets (*P* = 0.05).

**Table 4:** Effect of treatments with abamectin aqueous solutions at different concentrations on *Globodera pallida* in a pot experiment.

Treatment	Dose (µg/mL or g/m^2^) ^c^	Application Time	Cysts/100 g soil		Eggs and J2/g Soil		Eggs and J2/cyst		Reproduction Rate *Pf/Pi*	
Untreated control	0	---	98 ± 9.1 ^a^	A ^b^	126 ± 10.8	A	130 ± 5.4	A	4.6 ± 0.4	A
Abamectin	4.5	2 wks after sowing	107 ± 4.8	A	126 ± 8.0	A	119 ± 9.8	A	4.6 ± 0.3	A
Abamectin	9.0	2 wks after sowing	101 ± 8.6	A	108 ± 7.4	A	111 ± 8.6	AB	3.9 ± 0.3	A
Abamectin	18.0	2 wks after sowing	20 ± 2.6	BC	13 ± 2.1	C	68 ± 5.3	C	0.5 ± 0.1	C
Abamectin	36.0	2 wks after sowing	18 ± 1.7	C	14 ± 1.6	BC	83 ± 9.7	BC	0.5 ± 0.1	C
Fosthiazate	0.3	At sowing	40 ± 3.6	B	40 ± 5.7	B	100 ± 8.9	AB	1.5 ± 0.2	B

^a^ Each value is an average ± SE of 16 replications from two independent experiments; ^b^ Data flanked in each column by the same letters are not statistically different according to the Least Significant Difference’s Test (< 0.01). ^c^ g/m^2^ is referred to the granular product fosthiazate.

**Table 5:** Percentage of hatching of eggs from *Globodera pallida* newly formed cysts collected from soil treated with different concentrations of abamectin aqueous solutions.

Treatment	Dose (µg/mL or g/m^2^) a.i.	Hatching (%)	
Untreated control	0	51.1 ± 5.2 ^a^	A ^b^
Abamectin	4.5	49.7 ± 2.3	A
Abamectin	9	34.2 ± 2.5	B
Abamectin	18	22.2 ± 1.2	C
Abamectin	36	4.6 ± 1.3	D
Fosthiazate	0.3	28.0 ± 3.0	BC

^a^ Each value is an average ± SE of 8 replications from two independent experiments. ^b^ Data flanked by the same letters are not statistically different according to Least Significant Difference’s Test (*P* < 0.01).

**Table 6:** Abamectin doses required to kill 50, 60, 70, 80, 90 and 99.9% vitality of *Globodera pallida* eggs inside newly formed cysts extracted from soil treated with different concentrations (4.5, 9, 18 and 36 μg/mL a.i.) of abamectin.

Percentage Mortality	Abamectin Lethal Doses (µg/mL a.i.)
50	14.4 (12.9–16.0) ^a^
60	17.2 (15.4–19.1)
70	20.9 (18.8–23.2)
80	26.3 (23.6–29.3)
90	36.0 (32.3–40.1)
99.9	131.3 (118.0–146.1)

^a^ Fiducial limits (*P* = 0.05).
